# Postoperative outcome of ambulatory dogs with intervertebral disc extrusion causing incontinence and/or tail dysfunction: 18 cases (2010‐2020)

**DOI:** 10.1111/jsap.13497

**Published:** 2022-03-23

**Authors:** R. Pfund, A. K. Forward, R. Fentem, A. Nagendran, A. R. Fraser, A. H. Crawford

**Affiliations:** ^1^ Department of Veterinary Clinical Science and Services, Royal Veterinary College University of London Hawkshead Lane, North Mymms Herts AL9 7TA UK; ^2^ Department of Neurology and Neurosurgery, Davies Veterinary Specialists Manor Farm Business Park Higham Gobion, Hitchin SG5 3HR UK; ^3^ Department of Veterinary Science, Small Animal Teaching Hospital University of Liverpool Neston, Cheshire CH64 7TE UK; ^4^ Department of Neurology and Neurosurgery Anderson Moores Veterinary Specialists Hursley Winchester SO21 2LL UK

## Abstract

**Objectives:**

To assess the recovery of urinary continence, faecal continence and tail function in ambulatory dogs with caudal lumbar intervertebral disc extrusion and to explore clinical factors that may be associated with recovery.

**Materials and Methods:**

Medical records from January 2010 to December 2020 were searched to identify ambulatory dogs undergoing surgical treatment for a caudal lumbar intervertebral disc extrusion causing urinary incontinence, faecal incontinence and/or tail dysfunction. Signalment, history, presenting clinical signs, neurological examination findings, diagnostic test results, treatment and outcome were recorded for all dogs.

**Results:**

Eighteen dogs with caudal lumbar intervertebral disc extrusion causing tail dysfunction, urinary and/or faecal incontinence were included. Urinary continence was recovered in 12 (86%) of 14 affected dogs, faecal continence recovered in nine (90%) of 10 affected dogs and tail function recovered in 13 (87%) of 15 affected dogs. Loss of tail nociception was recorded in three dogs on presentation; two made a full recovery and one showed mild persistent tail paresis.

**Clinical Significance:**

The prognosis for functional recovery of urinary continence, faecal continence and tail function in ambulatory dogs with caudal lumbar intervertebral disc extrusion following surgical treatment is good. Larger studies are needed to identify prognostic factors associated with failure of recovery.

## INTRODUCTION

Degenerative intervertebral disc disease is a common cause of neurological dysfunction in dogs, with an estimated lifetime prevalence of 3.5% reported in dogs under 12 years old (Bergknut *et al*. 2012). The intervertebral disc consists of the inner gelatinous nucleus pulposus (NP), the outer laminated anulus fibrosus, the transition zone and the cartilaginous end plates (Bergknut *et al*. 2013). Intervertebral disc extrusion (IVDE) is characterised by herniation of degenerate and calcified NP material through all layers of the ruptured annulus fibrosus into the vertebral canal (Hansen 1951, Smolders *et al*. 2013). The resulting clinical signs are caused by the compression and/or contusion of the overlying spinal cord and nerve roots and may include spinal pain, motor and sensory deficits. The cauda equina is the collection of sacral and caudal spinal roots and nerves that extend caudally through the vertebral canal, beyond the conus medullaris (or terminal spinal cord), to exit at their respective intervertebral foramina (Evans & de Lahunta 2013). Caudal lumbar IVDEs may compress the cauda equina and thus compromise innervation to the lower urinary tract, rectum, perineum and tail. Associated clinical signs may include urinary incontinence (UI), faecal incontinence (FI) and tail paresis/plegia, with preservation of pelvic limb motor function. These patients represent a unique subset of IVDE patients as, despite minimal impairment of pelvic limb function, failure to recover urinary and faecal continence has significant implications for the owner and the long‐term care needs of their pet. However, there is limited information available on the prognosis for recovery of urinary and faecal continence in dogs with caudal lumbar IVDEs. In thoracic and cranial to mid‐lumbar IVDE, the prevalence of postoperative urinary and FI in dogs presenting with paraplegia and intact nociception has been reported as 8 and 4% respectively, compared to 37 to 41% and 18 to 41% in dogs presenting with paraplegia and absent pelvic limb nociception (Olby *et al*. 2003, Bush *et al*. 2007, Aikawa *et al*. 2012). However, these patients had significant spinal cord injury and thus differ from the nerve root compression that would be expected with a caudal lumbar IVDE affecting the cauda equina. In a study of 36 dogs with caudal lumbar IVDE, the probability of regaining functional recovery (defined as independent ambulation, resolution of spinal pain, urinary and faecal continence) was comparable to dogs with thoracolumbar IVDE. However, 75% of these dogs were paraplegic on presentation and preoperative continence and tail function were not evaluated, but of the seven dogs that failed to make a functional recovery all had UI and/or FI (Dhupa *et al*. 1999). Given the inherently greater regenerative capacity of the peripheral nervous system when compared with the central nervous system (Cahjal 1928, Buss *et al*. 2004, Gordon 2016, Curcio & Bradke 2018), the prognosis for functional recovery following nerve root injury would be anticipated to be better compared with functional recovery following spinal cord injury. This case series aims to provide clinical information to guide clinician and owner decision making when managing dogs presenting with caudal lumbar IVDE causing UI and/or FI as a dominant clinical sign.

## MATERIALS AND METHODS

### Medical record search

The digital medical database at the Royal Veterinary College, Davies Veterinary Specialists, the University of Liverpool and Anderson Moores Veterinary Specialists were searched to identify dogs with a caudal lumbar IVDE with UI and/or FI and/or reduced tail function undergoing surgical treatment between January 2010 and December 2020. Searched terms used included “intervertebral disc disease,” “intervertebral disc extrusion,” “urinary incontinence,” “faecal incontinence”, “tail paresis” and “tail plegia.”

### Inclusion criteria

Dogs were included if they presented with UI/FI and/or decreased tail function as a primary problem, were subsequently diagnosed by MRI or CT scan with an IVDE between L4 and S1 according to previously published criteria (Olby *et al*. 2000, Gomes *et al*. 2016) and had surgical report confirmation of the diagnosis of IVDE. Cases were excluded if the medical records or imaging studies were incomplete. The study was approved by the Royal Veterinary College Ethics and Welfare Committee (URN SR2020‐0071).

### Data extracted

Information acquired from the medical records included signalment, presenting clinical signs, duration of presenting clinical signs, physical examination findings, neurological examination findings, treatment undertaken, duration of hospitalisation and outcome. Neurologic status was graded by the Modified Frankel Score (MFS) defined as paraplegia with absent deep nociception (grade 0), paraplegia with absent superficial nociception (grade 1), paraplegia with intact nociception (grade 2), non‐ambulatory paraparesis (grade 3), ambulatory paraparesis and ataxia (grade 4), spinal hyperesthesia only (grade 5) or normal/not applicable (no spinal pain or pelvic limb deficits) (Van Wie *et al*. 2013). Dogs with an MFS of 0 to 3 were excluded from further analysis as significant spinal cord injury was likely to confound the recovery of urinary/faecal continence and tail function. Tail paresis was considered to include reduced voluntary movement of the tail, reduced tail tone and/or low tail carriage. Tail plegia was defined as absence of voluntary movement in the tail.

Anaesthetic protocols varied between dogs but typically included premedication with a combination of 0.01 mg/kg intravenous acepromazine maleate and 0.1 to 0.2 mg/kg intravenous methadone, which was followed by anaesthetic induction with 4 to 6 mg/kg intravenous propofol and maintenance of anaesthesia with isoflurane or sevoflurane in oxygen. Low‐field (0.4 T) MRI was conducted at one institute, with high field (1.5 T) at the remaining three institutes. Dogs were placed in dorsal recumbency, and protocols included T2‐weighted (repetition time, 3000 milliseconds; echo time, 120 milliseconds) and T1‐weighted (repetition time, 400 milliseconds; echo time, 8 milliseconds) sagittal and transverse images. Slice thickness for sagittal and transverse images were typically 1.75 and 2.5 mm, respectively, with an interslice gap of 0.3 to 0.5 mm in both planes.

Surgical treatment consisted of a hemilaminectomy or dorsal laminectomy with removal of the extruded degenerate NP from the vertebral canal.

Follow‐up information was obtained from re‐examination visits 2 to 8.5 weeks following hospital discharge at each institute. Recovery was defined as regaining the ability to voluntarily urinate with a normal frequency/volume, defecate consciously and with a normal frequency/volume and return of normal tail function. Where cases showed persistent UI, FI or tail dysfunction at the re‐examination appointment further follow‐up information was sourced from the referring veterinary surgeon's clinical records.

## RESULTS

### Patient inclusion

Eighteen dogs met the study inclusion criteria and presented with a caudal lumbar IVDE causing UI and/or FI and/or tail paresis/plegia. Clinical data are summarised in Table 1.

**Table 1:** Signalment, clinical presentation, neurological examination findings, diagnosis, surgical treatment and outcome in 18 dogs with caudal lumbar IVDE causing UI, FI and/or tail dysfunction

Signalment	Bodyweight (kg)	Presenting clinical signs	Duration of clinical signs (day)	Neurological examination findings	MFS	UI	FI	Tail function	Neurolocalisation	Diagnosis	Surgical treatment	Time of follow‐up (days post discharge)	Outcome
5yo FN cocker spaniel	13.8	Progressive bilateral pelvic limb lameness. UI and FI	7	Mild bilateral pelvic limb lameness. Reduced withdrawal reflex in distal PLs. Reduced anal tone. Absent perineal reflex. Painful on caudal lumbar palpation	5	Y	Y	Plegia. Flaccid. Absent nociception	L6‐Caudal spinal cord segments or nerve roots	L5‐L6 IVDE	L5‐L6 right‐sided hemilaminectomy	36	Neurologically normal. UI and FI resolved
6yo ME pug	9.5	Progressive paraparesis. UI and FI	28	Ambulatory paraparesis. Delayed postural reactions PLs. Reduced anal tone. Absent perineal reflex	4	Y	Y	Paresis	L6‐Caudal spinal cord segments or nerve roots	L7‐S1 IVDE	L7‐S1 dorsal laminectomy	29	Mild paraparesis. UI improved but continued occasional urine dribbling. FI improved but occasional accidents in the house
6yo MN crossbreed	6.0	UI, FI. Tail paresis	6	Reduced withdrawal reflex in distal PLs. Absent perineal reflex. Muscle atrophy right PL	NA	Y	Y	Plegia. Flaccid	L6‐Caudal spinal cord segments or nerve roots	L5‐L6 IVDE	L5‐L6 left‐sided hemilaminectomy	60	Neurologically normal. UI and FI resolved
4yo ME French bulldog	13.3	Lethargy. Paraparesis. UI, FI	2	Ambulatory paraparesis. Delayed postural reactions PLs. Reduced withdrawal reflex in PLs. Reduced perineal reflex. Lumbar pain on palpation	4	Y	Y	Paresis. Reduced response to noxious stimuli	L6‐Caudal spinal cord segments or nerve roots	L4‐L5 IVDE	L4‐L5 right‐sided hemilaminectomy	35	Mild proprioceptive ataxia PLs. UI and FI resolved
6yo MN cocker spaniel	13.8	Progressive left‐lateralized paraparesis. Loss of tail function	5	Ambulatory paraparetic (worse on the left). Delayed to absent postural reactions PLs. Absent patellar and reduced withdrawal reflex left PL. Absent anal tone. Painful on lumbar palpation	4	Y	N	Plegia. Absent nociception	L4‐Caudal spinal cord segments or nerve roots	L5‐L6 IVDE	L4‐L6 left‐sided hemilaminectomy	31	Mildly delayed postural reactions left PL. Mildly decreased patella and withdrawal reflex left PL. Normal tail function and intact tail nociception. UI resolved
6yo MN Cavalier King Charles spaniel	8.9	Progressive paraparesis. Spinal pain. FI	2	Ambulatory paraparetic with proprioceptive ataxia of PLs. Delayed postural reactions PLs. Reduced withdrawal reflex in distal PLs. Caudal lumbar pain on palpation	4	N	Y	Paresis	L4‐Caudal spinal cord segments or nerve roots	L5‐L6 IVDE	L5‐L7 right‐sided hemilaminectomy	54	Ambulatory with mild paraparesis. Mildly delayed postural reactions in left PL. Reduced patellar reflex in left PL. FI resolved
3.5yo FN Cavalier King Charles spaniel	14	Spinal pain. Restlessness. Tail paresis	42	Normal	NA	N	N	Plegia. Reduced tone	Caudal spinal cord segments or nerve roots	L6‐L7 IVDE	L6‐L7 right‐sided hemilaminectomy	28	Neurologically normal. Normal tail function
9yo ME Cavalier King Charles spaniel	NR	Reluctance to jump progressing to UI and FI	14	Ambulatory paraparesis with mild proprioceptive ataxia PLs. Plantigrade. Delayed postural reactions PLs. Reduced withdrawal reflex PLs. Perineal reflex absent bilaterally. Reduced anal tone. Painful on caudal lumbar palpation	4	Y	Y	Paresis. Reduced tone	L4‐Caudal spinal cord segments or nerve roots	L5‐L6 IVDE	L5‐L6 right‐sided hemilaminectomy	32	Neurologically normal with resolution of UI and FI
10yo MN crossbreed	17.8	Tail paresis. UI. Pelvic limb ataxia	7	Ambulatory paraparetic. Proprioceptive ataxia of PLs. Delayed postural reactions PLs. Reduced perineal reflex bilaterally	4	Y	N	Plegia. Flaccid. Absent nociception	L4‐Caudal spinal cord segments or nerve roots	L6‐L7 IVDE	L6‐L7 right‐sided hemilaminectomy	59	Mild paraparesis and proprioceptive ataxia of PLs. Delayed postural reactions PLs. UI resolved. Mild tail paresis with reduced tone and tendency to hold tail to the left
6 yr MN cocker spaniel	16	Tail paresis. UI	2	Ambulatory paraparesis. Delayed postural reactions PLs. Reduced withdrawal reflex PLs. Reduced perineal reflex	4	Y	N	Paresis	L6‐Caudal spinal cord segments or nerve roots	L5‐L6 IVDE	L5‐L6 left‐sided hemilaminectomy	28	Ambulatory with mild paraparesis and proprioceptive ataxia of PLs. UI resolved. Mild tail paresis
11yo MN cocker spaniel	15	Spinal pain	28	Kyphotic. Intermittently delayed postural reactions PLs	5	Y	N	Normal	T3‐S3 spinal cord segments or nerve roots	L6‐L7 IVDE	L6‐L7 left‐sided hemilaminectomy	30	Neurologically normal. UI resolved
9yo MN Cavalier King Charles spaniel	7	Progressive paraparesis. Spinal pain	13	Ambulatory paraparesis. Delayed postural reaction PLs. Reduced withdrawal reflexes PLs. Absent perineal reflex. Absent anal tone. Pain on caudal lumbar and sacral palpation	4	Y	N	Normal	L6‐S3 spinal cord segments or nerve roots	L5‐L6 IVDE	L5‐L6 left‐sided hemilaminectomy	30	Neurologically normal. UI resolved
5yo ME Belgian Laekenois	33.5	Bilateral PL lameness. Yelping episodes. Reluctance to jump. Recent (7 days) loss of tail function	60	Reduced perineal reflex. Pain on caudal lumbar palpation	5	N	N	Paresis. Flaccid	S1‐Caudal spinal cord segments or nerve roots	L7‐S1 IVDE	L7‐S1 dorsal laminectomy	29 and 180	Neurologically normal
7y MN dachshund	5.4	Spinal pain. UI, FI. Low tail carriage	10	Reduced withdrawal reflex PLs. Pain on caudal lumbar palpation	5	Y	Y	Paresis	L6‐Caudal spinal cord segments or nerve roots	L5‐L6 IVDE	L5‐L6 right‐sided hemilaminectomy	21	Neurologically normal. UI and FI resolved
6yo MN dachshund	6.6	Reluctance to jump. Tail paresis	10	Painful on caudal lumbar spinal palpation	5	N	N	Paresis	Caudal spinal cord segments or nerve roots	L6‐L7 IVDE	L6‐L7 right‐sided hemilaminectomy	28	Neurologically normal
7yo MN dachshund	5.4	Progressive paraparesis. 2 to 3 days of UI	28	Ambulatory paraparetic. Reduced withdrawal reflexes PLs. Absent perineal reflex. Absent anal tone. Reduced perineal sensation	4	Y	Y	Paresis	L6‐caudal spinal cord segments or nerve roots	L5‐L6 IVDE	L5‐L6 right‐sided hemilaminectomy	14	Persistent UI. FI resolved. Re‐examination at RVS 90 days post discharge: persistent UI
7yo MN German shepherd dog	40.3	Low tail carriage. Intermittent difficulty urinating and urine dribbling. Straining when defaecating	28	Ambulatory paraparesis	4	Y	Y	Paresis	L6‐Caudal spinal cord segments or nerve roots	L7‐S1 IVDE	L7‐S1 dorsal laminectomy	62	Neurologically normal
1yo MN Pomeranian	5.5	Intermittent mild paraparesis. Spinal pain. Intermittent UI and FI	14	Spinal pain	5	Y	Y	Normal	S1‐S3 spinal cord segments or nerve roots	L7‐S1 IVDE	L7‐S1 dorsal laminectomy	28 and 180	Neurologically normal. UI improved (infrequent urine dribbling). FI resolved. Reassessed at 180 days: UI resolved

FN female neutered, MN male neutered, ME male entire, IVDE Intervertebral disc extrusion, UI Urinary incontinence, FI Faecal incontinence, MFS Modified Frankel Score, NA Not applicable, yo Years old, PL Pelvic limbs, Y Yes, present, L Lumbar, S Sacral, N No, absent, NR Not recorded, RVS Referring veterinary surgeon

### Signalment

Affected breeds were four (22%) Cavalier King Charles Spaniels, four (22%) cocker spaniels, three (17%) dachshunds, two (11%) crossbreeds, one (6%) French bulldog, Belgian Laekenois, Pug, German shepherd dog and Pomeranian. There were 16 (89%) males (12 neutered) and two (11%) females (both neutered). The median age was 6 years (range 1 to 11). The median bodyweight was 13.3 kg (range 5.4 to 40.3).

### Reasons for presentation

UI and/or FI were reported by the owners on presentation in 11 dogs, and abnormal tail function in eight. Additional presenting complaints included paraparesis in seven dogs, spinal pain in six, reluctance to jump in three and pelvic limb lameness in two. Twelve (67%) dogs had received medical therapy consisting of a non‐steroidal anti‐inflammatory medication (nine dogs) and/or gabapentin (eight dogs), prednisolone (one dog; Prednicare, Animalcare) or tramadol (one dog; Tralieve, Dechra) for a median of 7 days (range 1 to 14 days) before referral. The duration of clinical signs before referral ranged from 2 to 60 days, with a median of 10 days for those who recovered continence and tail function, and 28 days for those who did not recover.

### Neurological examination findings

Ten dogs were presented with ambulatory paraparesis (MFS 4), six (33%) dogs with spinal hyperesthesia (MFS 5) and two (11%) were presented with a normal gait tail and no spinal pain (recorded as MFS NA in Table 1). Fifteen (84%) dogs were presented with tail dysfunction; 10 with tail paresis and five with plegia. Loss of nociception in the tail was recorded in three (17%) dogs, each with tail plegia. Low tail carriage and/or reduced to absent tail tone was recorded in seven (39%) dogs. Assessment of anal tone and perineal reflex was recorded in 16 dogs; three dogs had reduced anal tone and three had absent anal tone. Four dogs had a reduced perineal reflex, and six had an absent perineal reflex.

### Diagnostic imaging findings

Seventeen (94%) dogs underwent MRI, and one underwent CT. The most frequently affected intervertebral disc space was L5‐L6 in nine dogs (50%), followed by L6‐L7 in four (22%) dogs, L7‐S1 in four (22%) dogs and L4‐L5 in one (6%) dog.

### Treatment

All dogs underwent surgical treatment. This consisted of hemilaminectomy [L4/L5 to L6/L7: 14 dogs (78%)] or dorsal laminectomy (L7‐S1: four dogs [22%]) at the affected disc space(s).

The median time from onset of clinical signs to surgery was 12 days (range 2 to 45), with a median duration of hospitalisation of 7 days (range 3 to 20).

### Outcome

Median time to initial follow‐up re‐examination was 30 days (range 14 to 62). Of 14 dogs presenting with UI, 12 (86%) recovered. Of 10 presenting with FI, nine (90%) recovered. Of 15 dogs presenting with tail paresis/plegia, 13 (87%) recovered with mild residual tail paresis documented in the remaining two. Eight (44%) dogs were presented with UI, FI and tail paresis/plegia, of which six (75%) recovered all functions, with a median time of follow‐up of 35 days (range 14 to 62 days). Of the three dogs with absent nociception in the tail recorded on presentation, one was presented with tail plegia, UI and FI and two with tail plegia and UI: two dogs recovered fully and one had mild residual tail paresis.

Two dogs failed to recover full continence. Both were presented with UI, FI and tail paresis; one dog had mild UI and FI (occasional accidents in the house) 29 days post hospital discharge, and the other was found to have persistent UI 90 days post hospital discharge. Both dogs had shown clinical signs for ~28 days before presentation. Two dogs showed persistent tail paresis 28 and 59 days post hospital discharge (one was presented with UI and tail paresis, the other with UI, tail plegia and absent nociception in the tail). These two dogs had shown clinical signs for 2 and 7 days before presentation.

## DISCUSSION

This case series reports the postoperative outcome in 18 dogs presented with UI, FI and/or tail paresis/plegia caused by caudal lumbar IVDE. Fourteen (78%) dogs recovered fully within 8.5 weeks of surgery. Two (11%) dogs showed residual incontinence on re‐examination 29 (UI and FI) and 90 days (UI only) following hospital discharge, while two dogs showed mild tail paresis 28 and 59 days post hospital discharge. Despite the small case number, this suggests that the prognosis for recovery of continence and tail function is good with surgical treatment of caudal lumbar IVDE.

Cocker spaniels and Cavalier King Charles spaniels appeared to be over‐represented, accounting for eight of 18 (44%) dogs in this case series. In a previous study, caudal lumbar IVDEs were significantly more common in English cocker spaniels compared with dachshunds (Cardy *et al*. 2016). English cocker spaniels with a caudal lumbar IVDE were found to have a longer duration of clinical signs before referral and more commonly presented with spinal hyperesthesia or unilateral pelvic limb lameness as the main clinical sign compared with those that had thoracolumbar or mid‐lumbar IVDEs. However, continence and tail function were not specifically evaluated (Cardy *et al*. 2016).

Loss of all three evaluated functions (urinary continence, faecal continence and tail function) may represent a more severe or extensive cauda equina injury. Interestingly, the recovery rate was 75% in dogs presenting with UI, FI and tail paresis/plegia, compared with 100% in three dogs presenting with tail paresis/plegia only. Mechanisms of cauda equina injury in dogs with IVDE may include compression, contusion and compromised blood supply. Severity of nerve injury can be categorised according to Seddon's classification, which considers the affected anatomical components of the nerve, the severity of their compromise and the associated potential for recovery (Seddon 1943). Neurapraxia, in which the nerve remains structurally intact but temporary conduction block causes motor and sensory deficits, is typically associated with blunt trauma and/or traction injuries and recovery of function is rapid (a few days to 12 weeks). Axonotmesis is characterised by disruption of the axon with retention of intact surrounding connective tissues and typically results in Wallerian degeneration followed by axonal regrowth. The time course of recovery is highly dependent on the length over which regeneration is required (approximately 1 mm/day) (Yan *et al*. 2011, Chhabra *et al*. 2014). Finally, neurotmesis, in which both the axon and surrounding connective tissue are compromised, results in a permanent loss of function unless prompt surgical intervention is undertaken (Chhabra *et al*. 2014). Neurapraxia would be suspected in the majority of dogs in this case series given their high likelihood and relatively short time frame of recovery. Furthermore, both compressive and contusive injuries induced by IVDE would be expected to cause conduction block and compromise vascular supply, without transection of the spinal roots and nerves, again most consistent with neurapraxia.

Surgical management of IVDE is typically advised in cases presenting with severe or progressive neurological deficits to enable access to and removal of the extruded disc material and so decompress the affected region of spinal cord and/or nerve roots (Olby *et al*. 2003, Brisson 2010, Langerhuus & Miles 2017, Jeffery *et al*. 2018, Moore *et al*. 2020). Surgical management of dogs with thoracolumbar IVDE that retain nociception in the pelvic limbs is associated with a successful outcome in over 90% of cases, while in more severely affected cases (in which nociception is lost in the pelvic limbs), a successful outcome is reported in 25 to 76% (Olby *et al*. 2003, Ito *et al*. 2005, Laitinen & Puerto 2005, Jeffery *et al*. 2016, Fenn *et al*. 2017). To date, no studies have evaluated if surgical decompression and restoration of perfusion to compressed spinal roots and nerves facilitates a more timely and complete functional recovery in dogs presenting with caudal lumbar IVDE. Interestingly, early studies demonstrated that spinal roots are more susceptible to compression block than peripheral nerve, and more readily yield to tensile stress, potentially due to their lack of perineurium (Sunderland & Bradley 1961, Sharpless 1975). Thus, surgical intervention may alleviate conduction block induced by compression of the nerve root by the extruded disc material. In a study of degenerative lumbosacral stenosis, in which chronic compression of the cauda equina can result in a range of clinical signs including spinal pain, paraparesis, UI, FI and tail paresis, 11 dogs had UI on presentation, of which five recovered. Median duration of UI before presentation was 0.5 months in the dogs that recovered, compared with 2 months for those that did not. The probability of a poor outcome was found to be 5.88 times higher for those dogs with UI of ≥1 month, compared with dogs with UI of <1 month (De Risio *et al*. 2001). In our case series, the median duration of clinical signs for dogs who recovered continence was 10 days, compared with 28 days for the two dogs that did not recover incontinence. Thus, chronicity of cauda equina compression may contribute to likelihood of recovery but further studies are needed to investigate this.

Sacrocaudal luxation (or tail pull injury) in cats is associated with traumatic traction of the cauda equina. Intact tail base sensation, perineal reflex and anal tone have been shown to be positive prognostic indicators in cats with sacrocaudal luxation, with 75 to 100% of cats regaining urinary continence within 1 month of injury (Smeak & Olmstead 1985, Tatton *et al*. 2009). Loss of tail base sensation, perineal reflex and anal tone were associated with poorer functional outcome; 50 to 60% of affected cats recovered urinary continence within 30 days (Smeak & Olmstead 1985, Tatton *et al*. 2009, Couper & de Decker 2019). In the current study, three (16%) dogs were presented with loss of nociception in the tail; all three went on to make a functional recovery (mild residual tail paresis was reported in one dog). In the two dogs that failed to recover UI or UI and FI, reduced to absent anal tone and absent perineal reflex were documented on presentation. However, a further five dogs documented to have absent anal tone and/or perineal reflex on presentation went on to make a full recovery of continence and/or tail function. Thus, loss of anal tone, perineal reflex and tail nociception do not preclude a functional recovery. Further, larger scale studies are needed to investigate the prognostic utility of tail nociception, anal tone and perineal reflex in dogs with caudal lumber IVDE.

Limitations of this study include the small case number, multi‐institutional collaboration with associated variations in imaging and management protocols and the inevitable limitations inherent in a retrospective study, such as inconsistent data recording. Variables such as neurological status and time frame of neurological recovery are subjective and influenced by the clinician(s) managing the case. The MFS used is a subjective assessment of neurological status with scores ranging from 0 to 5 (Van Wie *et al*. 2013) and, while this scale has been previously validated, it has limited sensitivity as animals with considerable differences in the severity of their neurological deficits can be grouped together and it does not incorporate evaluation of tail function nor continence. Important clinical variables such as presence of tail base sensation, perineal sensation, anal tone, tail tone and ease of bladder expression were not recorded for all cases and so we are unable to determine whether these parameters were found to be normal and hence not reported (three of the collaborating institutes in this study typically record deficits only and not normal findings) or whether these variables were not assessed. Furthermore, the results of anal tone and perineal reflex were recorded as reduced or absent, which remain somewhat subjective and there may be differences in assessment method and interpretation between institutions and clinicians. Future prospective studies with careful and consistent evaluation of tail function/sensation, perineal reflex, perineal sensation, anal tone and bladder expression are likely to be highly informative and may enable identification of clinical variables associated with prognosis. While dogs with paraplegia or non‐ambulatory paraparesis were excluded to attempt to limit our case series to dogs with cauda equina compression rather than spinal cord injury, it is likely that some included cases had both spinal cord and cauda equina injury. Regardless, our data support a high likelihood of recovery from UI, FI and tail paresis and may be more representative of the typical clinical scenario in which extruded disc material can cause multilevel or extensive compression of the vertebral canal contents. A further limitation of this study was that follow‐up times were limited, and the timing of reassessments varied amongst patients. A more detailed and consistent assessment of recovery and neurological grade at specific time points after hospital discharge would facilitate a more detailed evaluation of long‐term outcome. Longer follow‐up might also have identified improvement in the dogs showing residual UI or tail paresis. All cases in this study underwent surgical treatment; future studies to evaluate outcome with medical treatment would provide a useful comparison. Finally, all cases presented in this study were referred to a veterinary specialist. Referred cases may be more severely affected, and/or may have more motivated owners and therefore may not be truly representative of the spectrum of IVDE cases in the canine population. In future studies, inclusion of a more varied case load, including those from first opinion practises would be worthwhile.

In this case series, we report the clinical presentation and outcome of 18 ambulatory dogs presenting with UI and/or FI and/or tail dysfunction as a result of caudal lumbar IVDE. All dogs underwent surgical management following which 14 made a full recovery, two dogs showed persistent UI and/or FI and two showed persistent mild tail paresis. Thus, overall the prognosis for recovery of tail function and continence is good in dogs with caudal lumbar IVDE undergoing surgical treatment. Further larger scale studies are needed to identify prognostic factors associated with failure of recovery of continence and tail function.

### Conflict of interest

None of the authors of this article has a financial or personal relationship with other people or organisations that could inappropriately influence or bias the content of the paper.
